# Waste anesthetic gases have a significant association with deoxyribonucleic acid (DNA) damage: A systematic review and meta-analysis of 2,732 participants

**DOI:** 10.1016/j.heliyon.2023.e19988

**Published:** 2023-09-09

**Authors:** Mayang Indah Lestari, Krisna Murti, Iche Andriyani Liberty, Zen Hafy, Violantina Linardi, Muhammad Khoirudin, Tungki Pratama Umar

**Affiliations:** a Doctoral Study Program in Biomedical Science, Faculty of Medicine, Universitas Sriwijaya, Palembang, Indonesia; b Department of Anesthesiology and Intensive Therapy, Faculty of Medicine, Universitas Sriwijaya, Dr. RSUP. Mohammad Hoesin Palembang, RS. Siti Fatimah, Palembang, Indonesia; c Department of Anatomical Pathology, Faculty of Medicine, Universitas Sriwijaya-RSUP Dr. Mohammad Hoesin, Palembang, Indonesia; d Department of Public Health, Faculty of Medicine, Universitas Sriwijaya, Palembang, Indonesia; e Department of Anesthesiology and Intensive Therapy, Faculty of Medicine, Universitas Sriwijaya, Palembang, Indonesia

**Keywords:** Waste anesthetic gases, Comet assay, Micronuclei, Chromosomal aberration

## Abstract

**Introduction:**

Operating room workers are at risk of experiencing adverse effects due to occupational exposure to waste anesthetic gases (WAGs). One of the consequences of long-term WAGs exposure is the probability of developing deoxyribonucleic acid (DNA) damage. This systematic review investigated the link between WAGs and DNA damage in operating room workers.

**Methods:**

PubMed, Science Direct, ProQuest, Scopus, and EbscoHost, as well as hand-searching, were used to find literature on the relationship between WAGs and DNA damage. Three independent reviewers independently assessed the study's quality. Meta-analysis was conducted for several DNA damage indicators, such as comet assay (DNA damage score, tail's length, tail's DNA percentage), micronuclei formation, and total chromosomal aberration.

**Results:**

This systematic review included 29 eligible studies (2732 participants). The majority of the studies used a cross-sectional design. From our meta-analysis, which compared the extent of DNA damage in operating room workers to the unexposed group, operating room workers exposed to WAGs had a significantly higher DNA damage indicator, including DNA damage score, comet tail's length, comet tail's DNA percentage, micronuclei formation, and total chromosomal aberration (p < 0.05) than non-exposed group.

**Conclusion:**

Waste anesthetic gases have been found to significantly impact DNA damage indicators in operating room personnel, including comet assay, micronuclei development, and chromosomal aberration. To reduce the impact of exposure, hospital and operating room personnel should take preventive measures, such as by adapting scavenger method.

## 1 Introduction

Waste anesthetic gases (WAGs) are a small amount of anesthetic gas, both nitrous oxide (N_2_O) and halogen anesthetics (such as halothane, enflurane, isoflurane, and desflurane), which leak from the patient's breathing circuit into the operating room air during the administration of anesthesia [1]. The WAGs have the potential to endanger health workers in hospitals such as anesthesiology specialists, nurse anesthetists, surgeons, operating room nurses, operating room technicians, and other operating room personnel [[2], [3], [4]]. Its impact can be classified into two categories: short-term (fatigue and lethargy) and long-term exposure (related to many disorders, both for the workers and fetuses) [5,6].

Operating room workers can be exposed to WAGs, even if the scavenging and ventilation systems are properly installed as a result of leaks through anesthetic gas delivery systems during system disconnections, from facemask connections or endotracheal tubes, or during induction of anesthesia [6,7]. Exposure is most common in health facilities that are not equipped with scavenging or ventilation systems or are equipped but in poor condition [1,2]. The United States Regulatory Agency, Occupational Safety and Health Administration (OSHA), estimates as many as 200,000 health workers are at risk of having an occupational disease due to chronic exposure to WAGs [3,8].

Chronic exposure to WAGs may harm the genetic composition, including causing deoxyribonucleic acid (DNA) damage [9]. It can elevate the risk of developing chronic illnesses like cancer, liver problem, and kidney disease. Furthermore, congenital defects, preterm deliveries, spontaneous abortions, and infertility can also arise following long-term exposure to WAGs [6]. Nonetheless, volatile anesthetics have been classified as group 3 (not classifiable as carcinogenic) by the International Agency for Research on Cancer (IARC) as long as exposure stays within the permissible range [10]. Assessment of WAGs is still crucial because nearly half of the operating rooms remain functioning without scavenging devices, particularly in less-developed nations, posing excessive and chronic exposure to WAGs that can lead to detrimental effects in humans [9].

Human biomonitoring is needed to evaluate genetic and chromosomal damage in individuals exposed to genotoxic substances [11,12]. Technological developments have made it possible to diagnose genetic disorders down to the molecular level. Comet assays (CA) are recognized for their robustness, sensitivity, and statistical power to evaluate deoxyribonucleic acid (DNA) cleavage [13]. Meanwhile, micronucleus assay examination, especially the assessment in buccal epithelial cells, can detect mutagenicity biomarkers, which are preferred to be used instead of chromosomal aberration tests because they do not require karyotype analysis and cell cultures, while also fast and inexpensive [14,15]. Due to the potential impact of inhalational anesthetics and genetic problems, we conducted a systematic review to analyze the association between WAGs and DNA damage in operating room workers.

## 2 Methods

The researchers conducted a literature search across multiple databases to gather publications on the impact of WAGs exposure to DNA damage. This review was established using the Preferred Reporting Items for Systematic Reviews and Meta-Analysis (PRISMA) 2020 guidelines [16]. This study's protocol has been registered to PROSPERO (CRD42022382476).

### 2.1 Eligibility criteria

This review focused on publications about healthcare workers' exposure to WAGs (measured by comet assay, micronuclei formation, and total chromosomal aberration) and their DNA damage asessment in the human operating room landscape. We included observational studies that used a standardized examination method (prospective or retrospective cohort, case-control, or cross-sectional study) and employed study participants aged over 18 years old. They must be consisted of both exposed and non-exposed (control) groups. Conference abstracts, literature reviews, opinion pieces, protocols, case reports, case series, and unretrievable full texts were not considered. Studies conducted in veterinary hospitals were also excluded from our analysis. To ensure data precision, only full-text manuscripts published in English were included.

### 2.2 Search strategy

The electronic search was conducted in five databases: PubMed (29 hits), Science Direct (26 hits), ProQuest (24 hits), Scopus (38 hits), and EbscoHost (29 hits). The search was accomplished on January 8th, 2023, and studies published between 2002 and 2022 were included. Hand-searching was also carried out by manually reviewing the references of the selected papers to locate relevant publications that were not indexed in the previously observed records [17]. The titles and abstracts of the studies found through the database search were assessed, and only those that met the eligibility requirements were contemplated for further analysis. Table 1 contains a list of the keywords used in the investigation.

**Table 1:** Search strategy.

Search	Query	Results
**EbscoHost**	(“Anesthetic Gases” OR “waste anesthetic gases”) AND (“Anesthesiologists” OR “Operating Room Nurse” OR “Anesthesiology resident” OR “Operating room worker”) AND (“DNA Damage” OR “DNA Injury” OR “genetic damage” OR “genetic instability”)	29
**ProQuest**	(“Anesthetic Gases” OR “waste anesthetic gases”) AND (“Anesthesiologists” OR “Operating Room Nurse” OR “Anesthesiology resident” OR “Operating room worker”) AND (“DNA Damage” OR “DNA Injury” OR “genetic damage” OR “genetic instability”)	24
**Pubmed**	(“Anesthetic Gases” OR “waste anesthetic gases” OR “Nitrous Oxide” OR “halogen anesthetics” OR “halogen” OR “sevoflurane” OR “isoflurane” OR “desflurane”) AND (“Anesthetists” OR “Anesthesiologists” OR “Operating Room Nurse” OR “Anesthesiology resident” OR “Anesthetic Trainee” OR “Operating room personnel” OR “Operating room worker”) AND (“DNA Damage” OR “DNA Injury” OR “genetic damage” OR “genetic instability”)	29
**Science Direct**	(“Anesthetic Gases” OR “waste anesthetic gases”) AND (“Anesthesiologists” OR “Operating Room Nurse” OR “Anesthesiology resident” OR “Operating room worker”) AND (“DNA Damage”)	26
**Scopus**	(“Anesthetic Gases” OR “waste anesthetic gases” OR “Nitrous Oxide” OR “halogen anesthetics” OR “halogen” OR “sevoflurane” OR “isoflurane” OR “desflurane”) AND (“Anesthetists” OR “Anesthesiologists” OR “Operating Room Nurse” OR “Anesthesiology resident” OR “Anesthetic Trainee” OR “Operating room personnel” OR “Operating room worker”) AND (“DNA Damage” OR “DNA Injury” OR “genetic damage” OR “genetic instability”)	38

### 2.3 Study selection

The retrieved papers were inspected for potential duplication. Two reviewers (VL and MK) used Rayyan QCRI, a semi-automated abstract and title sorting program, to screen the titles and abstracts [18]. Inter-rater disagreements were resolved by careful re-examination and consultation of the paper among reviewers until a consensus was attained. The full texts of potentially eligible studies were acquired and independently evaluated by two reviewers (ZH and TPU) to determine eligibility for inclusion in the final analysis. The full-text screening stage used a similar method of resolving the disagreements among researchers. If no settlement could be actualized, a moderator (MIL) was present to re-evaluate the distinctions and finalize the manuscript inclusion designation.

### 2.4 Data extraction and quality assessment

The primary data extraction was performed by VL, MK, and TPU. The following data were extracted: authorship, country of research, study design, sample size (male/female), participants' age, occupation, body mass index (BMI), exposure period, anesthetic gas description (and concentrations when available), smoking, and alcohol consumption status. Two of the co-authors (KM and IAL) appraised the risk of bias of the included studies autonomously, with discrepancies resolved through mediation among researchers until a decision was attained. The Newcastle-Ottawa Scale (NOS) was used to evaluate every study's methodological quality. There are three sections in the NOS: selection, comparability, and outcome. It is graded using a star system distributed across three domains and then classified based on the level obtained as follows: high (0–3 stars), moderate (4–6 stars), or a low (7–9 stars) risk of bias [19]. For cross-sectional studies, the modified NOS follows a slightly different pattern, with low (7–8), moderate (5–6), and high (0–4) risk of bias [20].

### 2.5 Statistical analysis

Following the compilation of all included publications, the data were recorded in Microsoft Excel 2019 (version 2102). The I^2^ statistic was used to assess study heterogeneity, with the cut-off p < 0.1 and I^2^>50% considered as the evidence of considerable study heterogeneity [21]. Random-effects and fixed-effects meta-analysis were performed with a 95% confidence interval (CI) using Review Manager (RevMan) Version 5.4.1. (The Cochrane Collaboration). Meta-analysis was performed for each DNA damage indicator (DNA damage score/arbitrary unit, comet tail length, percentage of DNA in comet tail, micronuclei formation, and total chromosomal aberration). To be eligible for inclusion in the meta-analysis, studies had to report mean scores and standard deviations (SDs). However, if the central tendency was presented as a median or the data distribution was described as an interquartile range (IQR) or range, the calculation from Wan et al. [22] was used to convert it into desirable value. The standardized mean difference (SMD) method was applied in the meta-analysis to evaluate the impact of WAGs exposure on DNA damage. We extracted the value from the data presented as a diagram using WebPlotDigitizer version 4.6 (https://automeris.io/WebPlotDigitizer; Pacifica, California, USA).

### 2.6 Publication bias

Publication bias was examined utilizing funnel plots and Egger's linear regression test with Review Manager (RevMan) Version 5.4.1 and Comprehensive Meta-Analysis Version 3.3 (Biostat, Englewood, New Jersey). The presence of potential publication bias was indicated by an asymmetric distribution of data points in the funnel plot and a quantified result of p < 0.05 in the Egger's test. Asymmetry in the funnel plot was caused by factors other than publication bias, including minor study effects, heterogeneity, and chance, particularly in small sample size studies. Sensitivity analysis was performed by discarding each record incrementally to investigate the stability of the outcome. Meta-regression analysis was used to investigate the potential source of heterogeneity if a variable was observed by at least ten studies [23]. In the meta-analysis, all p-values were two-sided, and p < 0.05 was considered significant.

## 3 Results

### 3.1 Study characteristics

**Fig. 1:**
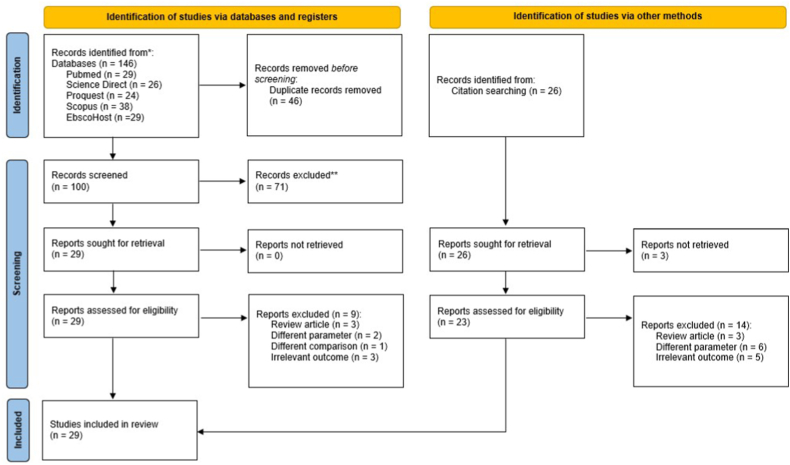
Study selection flow.

The search strategy identified a total of 172 studies (146 from registers and 26 from handsearching. At the final evaluation stage, 29 studies (2732 participants; 1405 in the exposed group and 1327 in the non-exposed group) were included (Fig. 1). Most of the studies were cross-sectional, with only three with case-control design [[24], [25], [26]] and one as the cohort study [4]. Most studies dominated by female, with 13 studies have a >50% proportion of male [24,[27], [28], [29], [30], [31], [32], [33], [34], [35], [36], [37], [38]]. Furthermore, smoking and alcohol consumption was reported in 16 and four studies, respectively, with an overall percentage of 33.92% smokers (326/961) and 36.54% alcoholics (95/260). Maximum exposure period is reported by El-Ebiary et al. [39], with 19.25 ± 2.36 years. Characteristics of the study population can be seen in Table 2.

**Table 2:** Population's characteristics.

ID	Author	Study Design	Country	Population (Exposed/Control)	Physician proportion (Exposed)	Age (Exposed)^a^	Exposure period (year)^a^	Gas Type	BMI	Gender (Exposed, Male/Total)	Smoking (Exposed, Yes/Total)	Alcohol (Exposed, Yes/Total)
1.	Aldrieny et al., 2013 [47]	Cross-sectional	Egypt	26/13	NA	31.19 ± 3.06	10.89 ± 1.93	H, I	NA	15/26	2/26	NA
2.	Baysal et al. (2009) [26]	Case-control	Turkey	30/30	NA	33 ± 5	7 ± 4	D, H, I, N, S	25 ± 5	19/30	NA	NA
3.	Bilban et al. (2005) [50]	Cross-sectional	Slovenia	153/153	153/153	NA	12.94 ± 6.52	H, I, N	NA	153/153	99/153	NA
4.	Borayek et al. (2018) [28]	Cross-sectional	Egypt	32/32	0/32	34.9 ± 6.5	17.75 ± 5.3	I	NA	0/32	NA	NA
5.	Braz et al. (2018) [40]	Cross-sectional	Brazil	30/30	30/30	28.5267 ± 1.61	3.06 ± 0.47	I, N, S	24.62 ±	18/30	NA	NA
6.	Braz et al. (2020) [9]	Cross-sectional	Brazil	31/32	NA	28.7 ± 1.9	3	I, N, S	24.6 ± 3.8	20/32	NA	NA
7.	Braz et al. (2020) [49]	Cross-sectional	Brazil	40/40	40/40	39 ± 14.3	3.5	I,S	25.5 ± 3.2	26/40	NA	NA
8.	Cakmak et al. (2019) [34]	Cross-sectional	Turkey	46/21	13/46	32.4 ± 5.7	NA	S	23.5 ± 3.2	9/46	21/46	4/46
9.	Chandrasekhar et al. (2006) [25]	Case-control	India	45/45	19/45	38.76 ± 8.66	10.468 ± 4.70	D, E, H, I, N, S, SP	NA	25/46	20/46	15/46
10.	de Araujo et al. (2013) [38]	Cross-sectional	Brazil	30/30	30/30	40.97 ± 11.25	13.83 ± 10.93	E, H, I, N, S,	NA	14/30	NA	NA
11.	El-Ebiary et al. (2013) [39]	Cross-sectional	Egypt	40/20	23/40	39.6 ± 6.32	19.25 ± 2.36	H, I, N, S	NA	25/40	14/40	NA
12.	Hua et al. (2021) [27]	Cross-sectional	China	68/82	NA	31.56 ±	8.29 ± 5.15	S	21.28 ±	19/68	4/68	6/68
13.	Izdes et al. (2010) [24]	Case-control	Turkey	40/40	0/40	36.8 ± 5.7	14.5 ± 6.6	D, I, N, S	NA	9/40	22/40	NA
14.	Kargar-Shouroki et al. (2019) [42]	Cross-sectional	Iran	60/60	10/60	36.17 ± 7.36	10.95 ± 5.58	I, N, S	20.75 ± 2.8	30/60	NA	NA
15.	Kargar-Shouroki et al. (2022) [35]	Cross-sectional	Iran	45/45	45/45	37.73 ± 6.91	12.36 ± 6.3	N	NA	19/45	5/45	NA
16.	Lewinska et al., 2005 [37]	Cross-sectional	Poland	46/28	0/46	42.9 ± 8.6	17.7 ± 10.1	I, N, S	NA	0/46	21/46	NA
17.	Musak et al. (2009) [29]	Cross-sectional	Czech Republic	76/76	41/76	36.89 ± 8.75	11.75 ± 9.35	NA	NA	15/76	23/76	NA
18.	Neghab et al. (2020) [41]	Cross-sectional	Iran	60/60	NA	36.17 ± 7.36	10.95 ± 5.58 ^b^	I, N, S	NA	30/60	NA	NA
19.	Paes et al. (2014) [4]	Cohort	Brazil	15/15	NA	27.9 ± 2.3	NA	I, N, S	25.5 ± 3.8	14/15	NA	NA
20.	Rozgaj et al. (2009) [33]	Cross-sectional	Croatia	50/50	20/50	38.88 ± 7.59	12.96 ± 8.96	NA	NA ± NA	12/50	16/50	NA
21.	Santovito et al. (2015) [48]	Cross-sectional	Italy	21/21	21/21	35.524	8.619 ± 4.364	NA	NA	15/21	NA	NA
22.	Shaker et al. (2011) [30]	Cross-sectional	Egypt	27/18	0/27	33.7 ± 7	15 ± 6.7	D, I, N, S	NA	0/27	0/27	NA
23.	Silva et al. (2022) [43]	Cross-sectional	Brazil	100/93	NA	34.2 ± 11.8	NA	I, N, S	25.5 ± 4.3	55/100	8/100	70/100
24.	Souza et al. (2016) [44]	Cross-sectional	Brazil	30/30	30/30	42 ± 15.9	NA	D, I, N, S	26.1 ± 3.3	20/30	NA	NA
25.	Souza et al. (2021) [45]	Cross-sectional	Brazil	30/30	30/30	NA	NA	H, N	26 ± 3	20/30	NA	NA
26.	Szyfter et al. (2016) [36]	Cross-sectional	Poland	100/100	26/100	NA	NA	NA	NA	15/100	24/100	NA
27.	Wiesner et al. (2008) [51]	Cross-sectional	Germany	14/14	14/14	32 ± 5	NA	S	NA	8/14	4/14	NA
28.	Wron'ska-Nofer et al. (2009) [31]	Cross-sectional	Poland	84/83	29/84	40.73	15.77	I, N, S	NA	29/84	39/84	NA
29.	Wronska-Nofer et al. (2012) [32]	Cross-sectional	Poland	36/36	0/36	NA	NA	I, N, S	NA	0/36	NA	NA

Results presented in mean ± standard deviation or mean (range).

Notes: *NA = Data Not Available, D = Desflurane, E = Enflurane, H = Halothane, I = Isoflurane, N = Nitrous oxide, S = Sevoflurane, SP = Sodium pentothal.

There are seven types of gases reported across the investigations in the operating room environment, including Isoflurane (20 studies), Sevoflurane (20 studies), Nitrous oxide (19 studies), Halothane (7 studies), Desflurane (5 studies), Enflurane (2 studies), and Sodium pentothal (1 study). Regrettably ten investigations [9,31,35,37,[40], [41], [42], [43], [44], [45]] found that the regular exposure limit for nitrous oxide (25 ppm time-weighted average/TWA) was exceeded the recommendation from the National Institute for Occupational Safety and Health (NIOSH), and six studies [9,40,41,43,44,46] found that the daily exposure limit for halogenated anesthetics (2 ppm) was breached. Meanwhile, fourteen studies did not report any information on WAGs concentration [4,[24], [25], [26],[28], [29], [30],^32^,^33^,^36^,^38^,^39^,^47^,^48^]. WAGs concentration are listed in Table 3.

**Table 3:** Concentrations (ppm) of WAGs in operating rooms.

	N_2_O (ppm)	Isoflurane (ppm)	Sevoflurane (ppm)	Desflurane (ppm)	Halothane
**Bilban et al. (2005)** [50]	0–100^b^	0–10^b^	–	–	0–10
**Braz et al. (2018)** [40]	155 ± 138	5.1 ± 4.2	9.8 ± 9.0	–	–
**Braz et al. (2020)** [9]	180 (61–350)^a^	5.3 (0.3–17.8)^a^	9.7 (1.0–34.1)^a^	–	–
**Braz et al. (2020)** [49]	–	1.25 ± 0.61^a^	1.74 ± 0.73^a^	–	–
**Cakmak et al. (2019)** [34]	–	–	0.427 (0.32–0.58)^a^	–	–
**Hua et al. (2021)** [27]	–	–	1.11 ± 0.65	–	–
**Lewinska et al. (2005)** [37]*	7.78–1282.13	–	–	–	–
**Neghab et al. (2020)** [41] **and Kargar-Shouroki et al. (2019)** [42]	850.92 (10–3895)^a^	2.4 (0.49–4.15)^a^	0.18 (0.01–0.59)^a^	–	–
**Kargar-Shouroki et al. (2022)** [35]	450.27 ± 327.44^a^	–	–	–	–
**Silva et al. (2022)** [43]	165 ± 15	7 ± 5	9 ± 7	–	–
**Souza et al. (2016)** [44]	150.3 ± 135.7	5.5 ± 4.4	7.7 ± 8.7	16.4 ± 6.0	–
**Souza et al. (2021)** [45]	150 ± 136	–	–	–	10 ± 6.4
**Wiesner et al. (2008)** [51]	–	–	0.2 (0.08–2.24)^c^	–	–
**Wron′ska-Nofer et al. (2009)** [31]*	244.43 (19.89–834.39)^a^	0.689 (0.066–1.855)^a^	0.574 (0.05–1.83)^a^	–	–
**Wron′ska-Nofer et al. (2012)** [32]*	102.77–834.39 ^b^	0.053–1.988 ^b^	0.061–1.711 ^b^	–	–

Note: *value presented as the conversion from mg/m^3^ using the formula: $\text{Concentration}\;\left(\text{ppm}\right)=\frac{24.45\;x\;\text{concentration}\;\left(\text{mg}/m3\right)}{\text{molecular}\;\text{weight}}$.

Data was presented in mean ± standard deviation except stated otherwise (^a^ Mean (range) ^b^ Range, ^c^ Median (range). Data was compiled only from studies which stated the gas concentration explicitly.

All studies evaluated the association of DNA damage with waste anesthetics gases (WAGs) in operating room workers. DNA damage was analyzed using three methods, comet assay and micronuclei formation assay (buccal and lymphocyte), and total chromosomal aberration. The comet assay was determined as DNA damage score (arbitrary unit) [4,9,26,[31], [32], [33],^44^], percentage of DNA in comet tail [24,39,45], and comet tail length [25,33,36,39]. Meanwhile, studies that carried out micronuclei formation assay was divided into two groups, namely buccal [9,25,34,40,43,44,49] and lymphocyte micronuclei [[33], [34], [35],^37^,^38^,^41^,^50^,^51^]. Chromosomal aberrations are also reported in eight studies [25,[28], [29], [30],^42^,^47^,^48^,^50^]. Other parameters are γH2AX/β-actin ratio [27] and relative telomere length [45,49].

### 3.2 Meta-analysis on impact of anesthetic gas exposure to comet assay, micronuclei formation, and chromosomal aberration

**Fig. 2:**
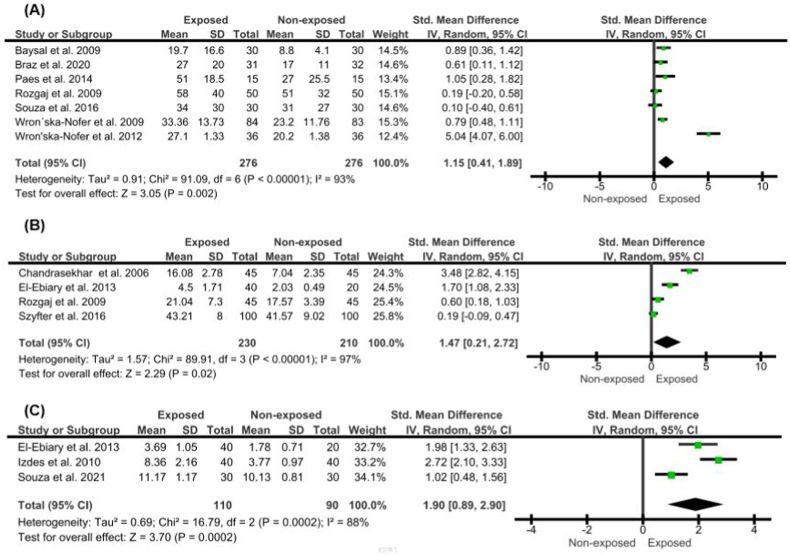
Effect of WAGs exposure to (A) Comet's assay arbitrary unit, (B) Comet's tail length, (C) %Tail DNA. The arbitrary unit was displayed as a weight-averaged degree of DNA breakage (between 0 and 400), tail length was determined in micrometers (μm), and %tail DNA was examined utilizing a computerized image evaluation system.

**Fig. 3:**
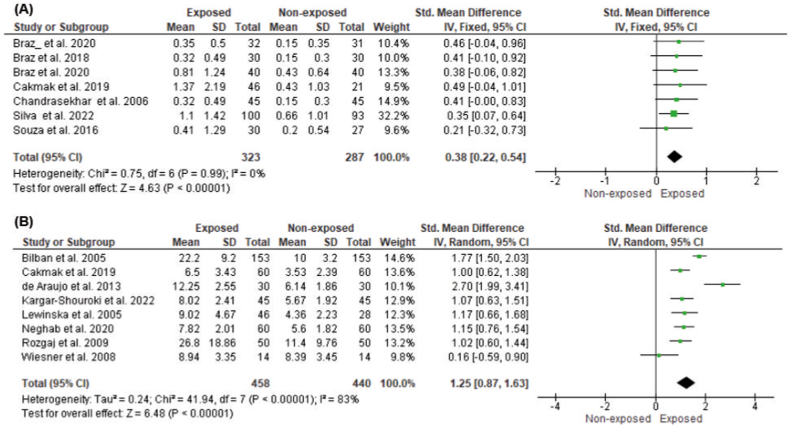
Effect of WAGs exposure to (A) Micronuclei (buccal), (B) Micronuclei (lymphocyte). Data was presented per 1000 cells.

**Fig. 4:**
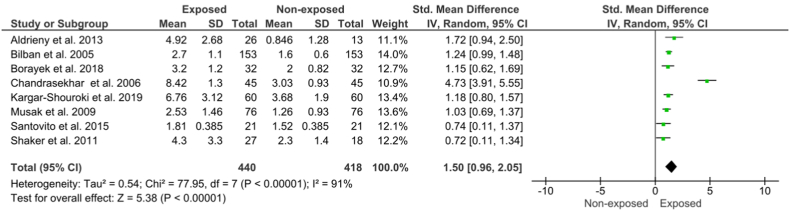
Effect of WAGs exposure to total chromosomal aberration. Data was counted per 100 metaphases cells.

The pooled mean results and 95% CI of the comet assay, micronuclei formation, and chromosomal aberration are presented in Fig. 2 (A - C), Fig. 3 (A, B), and Fig. 4, respectively. All studies have significant heterogeneity (I^2^>50%, p < 0.1), except for the analysis of buccal micronuclei; thus, random effect size determination was selected (for buccal micronuclei, fixed-effect meta-analysis was conducted). Comet assay examination in exposed individuals showed a significant difference from the non-exposed controls, either using DNA damage score (arbitrary unit) (pooled SMD = 1.15, 95% CI = 0.41–1.89; p = 0.002), tail's length (pooled SMD = 1.47, 95% CI = 0.21–2.72; p = 0.02), and percentage of DNA in comet tail (pooled SMD = 1.90, 95% CI = 0.89–2.90; p = 0.0002). Similar trends were also observed in buccal micronuclei formation (pooled SMD = 0.38, 95% CI = 0.22–0.54; p < 0.00001), lymphocyte micronuclei (pooled SMD = 1.25, 95% CI = 0.87–1.63; p < 0.00001), and total chromosomal aberration (pooled SMD = 1.50, 95% CI = 0.96–2.05; p < 0.00001).

### 3.3 Quality assessment

The Newcastle-Ottawa Scale (NOS) was used to determine the risk of bias. Two case-control and cohort studies received high-quality ratings (7–9), while two others received an intermediate grade (4–6). The NOS instrument was modified to make it more applicable for cross-sectional studies. Ten of the 25 studies (40%) were having low risk of bias (score 7–8), while the others (15/25; 60%) were having a moderate risk of bias. The total rating scores for the included studies ranged from 5 to 8 (mean: 6.36 ± 1.20; cross-sectional) and 4 to 7 (mean: 5.75 ± 1.09; case-control and cohort). Table 4 summarizes the quality of the included studies.

**Table 4:** Risk of bias analysis.

CROSS SECTIONAL	Selection	Comparability	Outcome	Total Score	Interpretation (Risk of Bias)
Aldrieny et al., 2013 [47]	***	0	**	5	Moderate
Bilban et al. (2005) [50]	****	0	*	5	Moderate
Borayek et al. (2018) [28]	****	0	*	5	Moderate
Braz et al. (2018) [40]	****	0	**	6	Moderate
Braz et al. (2020) [9]	****	0	**	6	Moderate
Braz et al. (2020) [49]	****	*	**	7	Low
Cakmak et al. (2019) [34]	****	**	**	8	Low
de Araujo et al. (2013) [38]	****	*	*	6	Moderate
El-Ebiary et al. (2013) [39]	****	0	*	5	Moderate
Hua et al. (2021) [27]	****	0	*	5	Moderate
Kargar-Shourouki et al. (2019) [42]	****	**	**	8	Low
Kargar-Shourouki et al. (2022) [35]	****	*	*	6	Moderate
Lewinska et al., 2005 [37]	****	**	*	7	Low
Musak et al. (2009) [29]	****	0	*	5	Moderate
Neghab et al. (2020) [41]	****	**	**	8	Low
Rozgaj et al. (2009) [33]	****	0	**	6	Moderate
Santovito et al. (2015) [48]	****	0	**	6	Moderate
Shaker et al. (2011) [30]	****	0	*	5	Moderate
Silva et al. (2022) [43]	****	**	**	8	Low
Souza et al. (2016) [44]	****	*	*	6	Moderate
Souza et al. (2021) [45]	****	**	**	8	Low
Szyfter et al. (2016) [36]	****	**	**	8	Low
Wiesner et al. (2008) [51]	****	**	**	8	Low
Wron'ska-Nofer et al. (2009) [31]	****	0	*	5	Moderate
Wronska-Nofer et al. (2012) [32]	***	**	**	7	Low
CASE CONTROL/COHORT	**Selection**	**Comparability**	**Outcome**	**Total Score**	**Interpretation (Risk of Bias)**
Baysal et al. (2009) [26]	**	0	**	4	High
Chandrasekhar et al. (2006) [25]	**	**	***	7	Low
Izdes et al. (2010) [24]	**	*	***	6	Moderate
Paes et al. (2014) [4]	***	0	***	6	Moderate

### 3.4 Publication bias

**Fig. 5:**
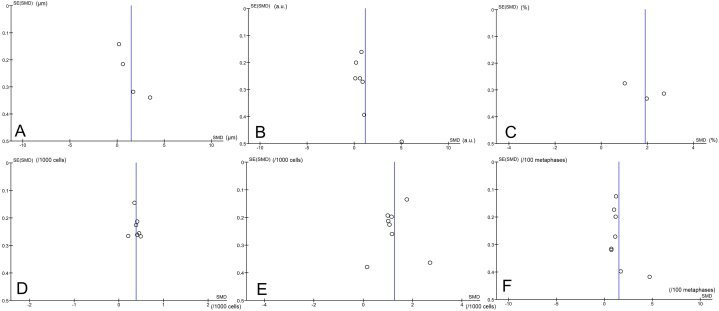
Funnel plot for the (A) comet tail length, (B) comet assay/DNA damage score (arbitrary unit), (C) %tail DNA, (D) buccal micronuclei, (E) lymphocyte micronuclei, (F) total chromosomal aberration. Y-axis (SE(SMD)) is Standard Error of Standardized Mean Difference, while X-axis is SMD. Abbreviation: a.u. = arbitrary unit.

The funnel plot and Egger's linear regression test were used to demonstrate publication bias. From a visual inspection of the Funnel plot, only buccal micronucleus formation illustrates an asymmetric distribution of the pooled publication, indicating the possibility of publication bias (Fig. 5 (A-F)). The DNA damage indicators including DNA damage score (comet assay arbitrary unit), comet tail length, %tail DNA, lymphocyte micronuclei, and total chromosomal abbreviation revealed no publication bias (p > 0.05). However, there is a publication bias (p = 0.002) for buccal micronuclei based on Egger's test (Table 5). Then, we performed a sensitivity analysis based on the comparability and outcome quality assessment. It was demonstrated that there was no significant change, denoting that the finding of the buccal micronuclei meta-analysis was stable. Nonetheless, due to the small sample size (number of included studies) and high heterogeneity across all studies, it is difficult to conclude the existing publication bias based on the above assessments. Despite the significant heterogeneity, we did not conduct meta-regression because all variables were observed in fewer than ten studies.

**Table 5:** Tests for publication bias.

DNA damage indicator	Egger's test		
	t-value	95% CI	P-value
Comet assay (arbitrary unit)	1.787	−3.192 ─ 17.762	0.134
Tail length (μm)	0.653	−39.209 ─ 53.248	0.581
%Tail DNA	1.172	−234.494 ─ 282.149	0.450
Buccal micronuclei	5.489	4.465 ─ 12.332	0.002
Lymphocyte micronuclei	0.551	−27.657 ─ 42.742	0.605
Total chromosomal aberration	0.239	−10.445─ 12.704	0.819

## 4 Discussion

WAGs can have debilitating short- and long-term impacts on the health of individuals. Short-term exposure to WAGs can cause headaches, fatigue, nausea, drowsiness, impaired work productivity, and problems with judgment and coordination. On the other side, long-term exposure to WAGs is associated with an assortment of health issues, including nephrotoxic, neurotoxic, hepatotoxic, immunosuppressive, and reproductive toxicological effects. Additionally, WAGs exposure over an extended period may damage DNA [4,5,52].

There are several theories that support the role of oxidizing drug metabolism and anesthetics for generating reactive oxygen species (ROS) and direct damage to genomes in the cell cycle, nucleic acids, lipids, and proteins. The imbalance between the production of ROS and antioxidants is known as oxidative stress. Oxidative stress can cause damage to macromolecules, including nucleic acids, lipids, and proteins that cause cell damage, as well as various diseases [1,9]. Further understanding of the association between DNA damage and oxidative stress with WAGs is needed to prevent occupational diseases.

Mechanisms of genotoxicity and DNA damage from halogens anesthetics and N_2_O are still unclear. There are several hypotheses of DNA damage and one of them is that exposure to N_2_O can interfere with the synthesis of nucleic acids and proteins [53]. In addition, a series of stress responses can occur after DNA damage has occurred in cells. This stress response induces a signaling cascade and stops the cell cycle until the damage is repaired. One of the main components of the signaling cascade is histone variant H2AX, which can be phosphorylated when a DNA double-strand break (DSB) occurs and then initiates damage repair mechanisms. H2AX plays a very important role in the identification and repair process of DSB [54,55].

According to our systematic review, WAGs are linked to an array of DNA damage indicators. This connection is most evident in people who have experienced chronic WAG exposure over an average of three to nineteen years. The alteration of the body's endogenous antioxidant framework, which is essential in preventing genotoxicity, may be the cause of this relationship in conjunction with the potential direct genotoxic consequences stated previously. When compared to the non-exposed group, the WAGs-exposed group has increased lipid peroxidation, decreased antioxidant thiol groups and enzyme activity (particularly glutathione peroxidase and superoxide dismutase), and decreased antioxidant capacity [1]. This association was further supported by a study by Wronska-Nofer that showed a substantial correlation between the level of reactive oxygen species (ROS), nitrous oxide concentration, and cumulative DNA damage [32]. Furthermore, we also found that approximately half of the included studies have higher than recommended level of WAGs than the guideline announced by the 10.13039/100000125NIOSH, with the recommended daily exposure limit on the concentration of WAGs in the operating room to minimize risk of occupational exposure was 25 ppm for nitrous oxide (N_2_O) and 2 ppm for halogen anesthetics such as halothane, enflurane, isoflurane, desflurane, and sevoflurane [56]. The problem of WAGs level were found particularly in N_2_O [32] which exceeds the predetermined threshold [9,31,35,37,[40], [41], [42], [43], [44], [45]]. Thus, it is recommended to use a scavenging system in the operating room to reduce levels of anesthetic gas waste and prevent potential health problems [57] However, the application of this system is still difficult in the developing countries, so other preventive measures must be taken. Regular monitoring of operating room air quality is necessary to determine levels of exposure to WAGs and identify anesthetic gas leaks and anesthetic machine malfunctions are important [58]. In addition, fourteen studies did not record any concentration of WAGs, indicating a potential dearth of workplace WAG surveillance programs, which aim to reduce health risk by assessing work-related exposure to the WAG during operations by reviewing each anesthetic breathing device no less than once every two years [59].

Comet assay (CA), also known as single cell gel electrophoresis or microgel electrophoresis, was introduced to detect DNA damage in eukaryotic cells or decomposing tissues caused by radiation. CA has been used in various studies, such as genetic toxicology, biological monitoring, genotoxicity, molecular epidemiology, nutrigenomics, studies of DNA repair systems, evaluation of the genotoxicity of nanomaterials, evaluation of the DNA integrity of mesenchymal stem cells and spermatozoa [12,15]. Although the majority of studies in our systematic reviews indicate a significant association between WAGs and CA examination (DNA damage score, comet tail length, and the percentage of DNA in the comet tail), there is one research that presents contrasting results. Souza et al.'s research [44] reported no significant changes in the overall DNA damage score. This outcome could be explained by the lymphocytes ability to develop an adaptive response, including memory formation, after prolonged exposure. This adaptive response may enhance the lymphocytes' ability to resist the harmful effects of substances such as anesthetics [33,36].

Micronuclei (MN) are small chromatin-containing spherical bodies that are visible in the cytoplasm of the cell. MN forming is caused by DNA damage or genomic instability. MN can occur as a result of natural processes, such as metabolism or aging or it can be caused by many different environmental factors, harmful habits, and diseases. The micronucleus examination that is often carried out is the buccal micronucleus cytome assay and lymphocyte [14]. From this examination, it was found that the frequency of micronuclei in the exposed group was higher than in the unexposed group and statistically significant. In our systematic review, we discovered a single investigation that gave a distinct conclusion from the majority of the included studies. This particular study revealed no difference in micronucleus (MN) development between the group exposed to WAGs and the non-exposed group [51]. However, it is crucial to highlight that the study used volatile anesthetic doses that were considerably lower than the suggested limit (0.2 ppm). It has been established that MN accumulates due to prolonged high-level WAG exposure, not low-level exposure [60]. This is a real concern since increased micronuclei formation may be associated with early carcinogenic events [61].

Other parameters such as chromosomal aberrations showed significant differences between the exposed and unexposed groups. These events are associated with late stages of apoptosis and cell death, respectively, although the exact mechanism is unknown [62]. In addition, basal cells in the exposed group were lower than in the unexposed group. The proportion of basal cells and cells undergoing cell death in the buccal mucosa is an indication of the regenerative capacity of the tissue. If the proportion is low, the regenerative capacity of the tissue is also low so that it can cause accelerated aging [63]. In this specific parameter, all of the included studies showed similar pattern, with positive difference on the extent of chromosomal aberration.

This systematic review has several limitations. Most of the included study designs were cross-sectional, indicating a lack of evidence. Furthermore, several studies only used a low sample size (<30 in each group). More studies with prospective cohort designs and large sample sizes are expected in the future. Meanwhile, our study strengths include large research inclusion, more variable description, and exclusively doing the meta-analysis of observational studies as compared with a previous systematic review (without the meta-analysis) [64].

## 5 Conclusion

There is a clear association between exposure to WAGs and DNA damage. Although the pathway of WAGs-induced DNA damage is uncertain, precautionary measures should be implemented. Some preventive measures include assembling a sufficient scavenging system in the operating room, using low fresh gas flow, increasing intravenous anesthetics administration, and limiting or avoiding nitrous oxide use. Furthermore, antioxidant supplementation can be carried out by operating room personnel.

We did not examine the risk of cancer as the primary outcome (we simply looked at the extent of DNA damage). Furthermore, we are unable to offer any additional interpretations of the IARC classification (the explanation cites some evidence of an increase in human cancer-related deaths/incidence but no effect on animal populations with low-level of exposure). However, an increased rate of surgery worldwide may represent a higher and longer exposure to WAGs, which must be described in a future study using rigorous methods. Moreover, additional research can be directed to other possible causes of DNA deterioration in operating room personnel, such as ionizing radiation from surgical techniques like the spine and endovascular surgery that may have synergistic implications for genotoxicity. Ultimately, a systematic review of the relationship between WAGs and cancer is another option for the future to address the things we have not done yet.

## Funding

None.

## Author contribution statement

Mayang Indah Lestari: Conceived and designed the experiments; Performed the experiments; Analyzed and interpreted the data; Contributed reagents, materials, analysis tools or data; Wrote the paper.

Krisna Murti: Conceived and designed the experiments; Analyzed and interpreted the data; Wrote the paper.

Iche Andriyani Liberty: Conceived and designed the experiments; Analyzed and interpreted the data; Wrote the paper.

Zen Hafy: Conceived and designed the experiments; Wrote the paper.

Violantina Linardi: Conceived and designed the experiments; Performed the experiments; Contributed reagents, materials, analysis tools or data; Wrote the paper.

Muhammad Khoirudin: Performed the experiments; Wrote the paper.

Tungki Pratama Umar: Conceived and designed the experiments; Performed the experiments; Analyzed and interpreted the data; Contributed reagents, materials, analysis tools or data; Wrote the paper.

## Data availability statement

Data will be made available on request.

## Ethics approval

Not applicable.

## Declaration of competing interest

The authors declare that they have no known competing financial interests or personal relationships that could have appeared to influence the work reported in this paper.
